# Adoptive Immunotherapy With Engineered iNKT Cells to Target Cancer Cells and the Suppressive Microenvironment

**DOI:** 10.3389/fmed.2022.897750

**Published:** 2022-05-09

**Authors:** Gloria Delfanti, Paolo Dellabona, Giulia Casorati, Maya Fedeli

**Affiliations:** ^1^Experimental Immunology Unit, Division of Immunology, Transplantation, and Infectious Diseases, Istituto di Ricovero e Cura a Carattere Scientifico (IRCCS) San Raffaele Scientific Institute, Milan, Italy; ^2^Vita-Salute San Raffaele University, Milan, Italy

**Keywords:** NKT cells, CD1d, cancer immunotherapy, CAR, T cell receptor, adoptive cell therapy (ACT)

## Abstract

Invariant Natural Killer T (iNKT) cells are T lymphocytes expressing a conserved semi-invariant TCR specific for lipid antigens (Ags) restricted for the monomorphic MHC class I-related molecule CD1d. iNKT cells infiltrate mouse and human tumors and play an important role in the immune surveillance against solid and hematological malignancies. Because of unique functional features, they are attractive platforms for adoptive cells immunotherapy of cancer compared to conventional T cells. iNKT cells can directly kill CD1d-expressing cancer cells, but also restrict immunosuppressive myelomonocytic populations in the tumor microenvironment (TME) *via* CD1d-cognate recognition, promoting anti-tumor responses irrespective of the CD1d expression by cancer cells. Moreover, iNKT cells can be adoptively transferred across MHC barriers without risk of alloreaction because CD1d molecules are identical in all individuals, in addition to their ability to suppress graft vs. host disease (GvHD) without impairing the anti-tumor responses. Within this functional framework, iNKT cells are successfully engineered to acquire a second antigen-specificity by expressing recombinant TCRs or Chimeric Antigen Receptor (CAR) specific for tumor-associated antigens, enabling the direct targeting of antigen-expressing cancer cells, while maintaining their CD1d-dependent functions. These new evidences support the exploitation of iNKT cells for donor unrestricted, and possibly off the shelf, adoptive cell therapies enabling the concurrent targeting of cancer cells and suppressive microenvironment.

## Introduction

Natural killer T cells (NKT cells) were originally characterized in mice as T cells that express both a TCR and NK1.1 (NKR-P1a-c or CD161), a C-type lectin NK receptor (1–3). Invariant NKT (iNKT) cells (or type I NKT cells) express a semi-invariant αβ TCR, formed by an invariant TRAV11-TRAJ18 (4) rearrangement in mice, or the homologous invariant TRAV10-TRAJ18 chain in humans (5), paired with a limited set of diverse Vβ chains, predominantly TRBV1, TRBV29, or TRBV13 in mice (6) and TRBV25 in humans (5). The semi-invariant TCR recognizes exogenous and endogenous lipid Ags presented by the monomorphic MHC class I-related molecule CD1d (7). Exogenous lipid Ags include the prototypical α-Galactosylceramide (α-GalCer) (8) and a number of bacterial-derived Ags (9). In addition, one of the iNKT cell functional hallmarks is their avid autoreactivity upon recognition of stress-associated cell endogenous lipids (10–13).

iNKT cells undergo a distinct developmental pathway compared to T cells, leading to the acquisition of innate effector functions already in the thymus. Thymic iNKT cells indeed express markers usually upregulated by peripheral effector/memory T cells, such as CD44 and CD69, together with distinctive NK differentiation markers, such as NK1.1 (in some mouse genetic backgrounds, CD161 in humans), CD122 (the IL-2R/IL-15R β-chain), CD94/NKG2 and Ly49(A-J), and a broad spectrum of T_H_1/2/17 effector cytokines (6).

Once migrated in the periphery, iNKT cells form a tissue resident population that survey the cellular integrity and rapidly respond to local damage and inflammation, jump starting the reaction by cells of the innate and adaptive immune response. In mice, iNKT cells with specific T_H_1, T_H_2 and/or T_H_17 effector profiles differentially colonize peripheral organs, resulting in the accumulation of specialized functions (14), whereas in humans it is more difficult to identify functional subsets beyond the two main CD4^+^ T_H_0 and CD4^−^ T_H_1 (15).

Amid functions exerted by iNKT cells in tissues, their active participation in the immune surveillance against malignant transformation and tumor progression is particularly well-established, strongly supporting their use in cancer immunotherapy. In this review, we will outline the advantages of harnessing iNKT cells particularly for adoptive immunotherapy, also when compared to conventional αβ T cells, given that they: (i) Control the tumor microenvironment (TME); (ii) Can be redirected against cancer cells by engineering with a tumor-specific CAR or TCR while maintaining their intrinsic control of the TME; (iii) Are devoid of alloreactivity, being restricted for the monomorphic CD1d molecule, allowing their possible use off the shelf in a donor-unrestricted manner.

## Role of iNKT Cells in Anti-Tumor Immune Response

The role of iNKT cells in anti-tumor immune response first emerged with the demonstration that the systemic administration of IL-12 (16) or α-GalCer into tumor-bearing mice resulted in iNKT cell activation, in turn promoting anti-tumor CD8^+^ T and NK cell responses able to control tumor progression (17–22). iNKT cells were also shown to exert spontaneous immune surveillance (i.e. without exogenous Ag stimulation) against methylcholanthrene-induced sarcomas or different genetically engineered mouse tumor models of sarcoma, lymphoma, prostate adenocarcinoma, Chronic Lymphocytic Leukemia (CLL) and pancreatic adenocarcinoma (23–30). In these studies, iNKT cell-deficient mice generated by the deletion of the genes encoding the invariant TRAJ18 TCR chain (Jα18^−/−^) or CD1d (CD1d^−/−^) were challenged with methylcholanthrene or crossed with tumor-predisposing genetically engineered strains, resulting in earlier onset and higher incidence of cancer with reduced survival. Adoptive transfer of iNKT cells into Jα18^−/−^ tumor bearing mice restored the protection against cancer, in the absence of exogenous stimulation (23, 30). Of note, the main CD4^+^ and CD4^−^ iNKT cell subsets were not equally effective in tumor control: in fact, liver-derived CD4^−^ iNKT cells were found to be the main mediators of tumor immune surveillance *in vivo*, by sustaining a T_H_1-type CD8^+^ T and NK cell-mediated immune response *via* IFNγ production (23).

In humans, clinical studies reported reduced frequencies and functional impairment of iNKT cells in patients with a wide range of solid and hematological malignancies (31). A decreased number and/or frequency of circulating iNKT cells associated with poor overall survival in prostate cancer (32), head and neck squamous cell carcinoma (33), neuroblastoma (34) acute myeloid leukemia (AML) (35) and CLL (24). Conversely, high numbers of intra-tumoral or circulating iNKT cells correlated with a good clinical outcome and improved survival in colorectal cancer (36), neuroblastoma (37), periampullary adenocarcinoma (38) and hematologic malignancies (39, 40), whereas circulating iNKT cells often become functionally impaired in patients with progressing cancers. In prostate cancer and in oral cell squamous carcinoma, iNKT cells have a defective production of IFNγ, acquiring a T_H_2 biased cytokine profile (41, 42). This defect can be reverted *in vitro* by activating the patient-derived iNKT cells in the presence of IL-2 or IL-12 (41), or *in vivo* upon therapeutic administration of autologous dendritic cells (DCs) pre-loaded with α-GalCer (43, 44). Studies in cancer patients also showed that iNKT cells respond to chemotactic signals derived from tumor cells, or cells of the tumor microenvironment, and infiltrate different types of primary and metastatic solid tumors (30, 45). iNKT cells infiltration in neuroblastoma is associated to CCL2 expression on tumor cells and CCL20 producing-TAMs, and iNKT cells preferentially infiltrate tumors that express high levels of this chemokine (46, 47).

## Mechanisms of Tumor Control by iNKT Cells

iNKT cells can control tumor progression by direct or indirect mechanisms. They can directly recognize and kill CD1d-expressing malignant cells, which are mainly hematopoietic malignancies (45, 48, 49). The mechanisms of direct killing include perforin/granzyme B-mediated cytolysis, TNFα production and TRAIL- and FasL- dependent apoptosis (29, 50, 51). However, what tumor associated lipid antigen/s iNKT cells recognize on cancer cells is currently unknown. Malignant cells can synthetize ganglioside GD3 that can stimulate iNKT cells *in vivo* (52); yet, it is likely that additional stress-related lipid antigens may be produced by cancer cells and presented to iNKT cells. Also in human cancers CD1d is mainly expressed by the hematopoietic ones (45), whereas very few solid tumors are CD1d-positive (53–55). Furthermore, cancer cells can downregulate CD1d surface expression, thereby limiting direct recognition by iNKT cells (55–57), even though they have shown to kill by perforin and/or granzymes at least colon cancer cells also in a CD1d-independent manner (58). The mechanisms underlying CD1d downregulation or loss by cancer cells are largely unknown. β-2 microglobulin loss, which can occur in progressing tumors, could affect CD1d in addition to MHC-I expression, while epigenetic silencing of MHC-I gene expression might also affect CD1d gene epigenetic control. Nevertheless, the available experimental data suggest that iNKT cells mainly control tumors by indirect mechanisms that impact the tumor microenvironment. iNKT cells promote the anti-tumor immune response through the maturation of CD1d^+^ DCs and the secretion of IFNγ and IL-12, in turn leading to the activation of anti-tumor CD4^+^ and CD8^+^ T cells and of NK cells, which ultimately cooperate in the elimination of both MHC-positive and -negative cancer cells (32, 59). Furthermore, more recent evidence shows that iNKT cells efficiently reprogram immunostimulatory functions of the TME by modulating myelomonocytic populations.

## iNKT Cells Shape the TME

Cancer cells undergo a progressive selection process in which a continuous and reciprocal cross-talk with the surrounding non-malignant cells of the TME plays a fundamental role for their growth and spread. Cells of both adaptive and innate immune response infiltrate the tumor stroma accounting for a major proportion of the TME (60, 61). Tumor infiltrating effector T cells, which can recognize tumor associated antigens (TAA) and selectively eliminate cancer cells together with NK cells, are in a critical equilibrium with immunosuppressive CD4^+^CD25^+^Foxp3^+^ T_regs_, providing one known mechanism determining tumor control vs. progression (62). The TME contains also B cells that can associate to either control or promotion of tumor progression (63, 64) and, especially, myelomonocytic cells that are the most abundant tumor infiltrating leukocyte population, accounting for up to 50% of the tumor mass (65). Tumor associated macrophages (TAMs) may have either tumor-opposing or promoting functions (66). An over-simplified model identifies TAMs with pro-inflammatory, immunostimulatory anti-tumor functions (M1-like), opposed to TAMs with pro-angiogenic, immunoregulatory tumor-supporting ability (M2-like) (65, 67–73). Tumor associated neutrophils (TANs), like TAMs, can be differently activated to support tumor progression or enhance their antitumor functions (74, 75), ranging from a pro-inflammatory N1 to a suppressive N2 state (76). Tumor infiltrating Myeloid-derived suppressor cells (MDSC), which can be divided in polymorphonuclear (PMN) and monocytic (M) MDSCs (77), exert unequivocal tumor-promoting activity (78); and can influence virtually every type of cancer therapy, from chemo-radiation to immunotherapy (61). DCs are also critical for eliciting potent anti-tumor T-cell responses, and patients with higher migratory CD103^+^ DCs have significantly increased overall survival (79). Cancer cells have also the ability to recruit cells from nearby stroma: in different tumors, stromal cell composition can vary substantially, including fibroblasts, vascular endothelial cells, stellate cells, and adipocytes. These cells secrete factors influencing proliferation, invasion, metastasization, and angiogenesis, but also antitumour immunity and responsiveness to immunotherapy (80). Overall, the functional plasticity of the immune cells in the TME defines a potential Achilles heel of the tumor, because reprogramming a dysfunctional TME toward a tumor-opposing state, in combination with the most advanced therapies, could result in cancer control and possibly remission (81).

iNKT cells actively infiltrate tumors. In hepatocellular carcinoma patients, the frequency of iNKT cells among CD3^+^ intrahepatic lymphocytes was similar to that found in peripheral blood (0.133%), but the iNKT cell frequency in tumor infiltrating lymphocytes coming from matched samples was doubled (0.271%) (82). An immunohistochemistry study showed that a small number of Vα24^+^ NKT cells was detected in the normal colorectal mucosa (2.6 +/– 3.7 cells/5 HPF), whereas a remarkably higher number was found in colorectal adenocarcinomas (15.2 +/– 16.3 cells/5 HPF), acquiring an independent prognostic value for the overall and disease-free survival rates (36). Vα24-Jα18 TCR mRNA expression was also detected in 53% (34) or 57% (37) of neuroblastoma cases, which also showed an higher overall survival rate compared to the patients in which the iNKT cell TCR could not be detected (34, 37).

Despite their low numbers, iNKT cells are normal components of both mouse and human TMEs (24, 34, 47, 55), where they can efficiently reprogram tumor opposing state irrespectively of the CD1d expression by cancer cells, by restraining the immunosuppressive functions of myelomonocytic cells such as TAMs, MDSC and TANs populations (83–85), as summarized in Table 1.

**Table 1:** iNKT cell interactions in the tumor microenvironment.

**References**	**Cell type**	**Effect**	**Interactors**	**Model**
De Santo et al. (83)	MDSCs	Inhibition, reduction of number of MDSCs	CD1d-iTCR CD40-CD40L	Influenza A infection
Ko et al. (86)	MDSCs	Differentiation of MDSCs into functional DCs, and subsequent activation of tumor-specific CD8^+^ and CD4^+^ T cells and triggering of NK cell cytotoxicity	CD1d-iTCR	colon carcinoma
Lee et al. (87)	MDSCs	Inhibition, reduction of number of MDSCs	CD1d-iTCR	Her-2/*neu*^+^ transfectoma EL4 thymoma
De Santo et al. (84)	Neutrophils	Reduction in IL-10 secretion by neutrophils and increase in IL-12 production; reduction of neutrophil immune suppression	CD1d-iTCR CD40-CD40L	melanoma
Song et al. (85)	TAMs	Elimination and impairment of TAM tumor-supporting activity	CD1d-iTCR	humanized neuroblastoma
Liu et al. (47)	TAMs	iNKT cells are recruited in the TME tumor by CCL20-producing TAMs, and become progressively inhibited by macrophage-induced hypoxia	Secreted CCL20-CCR6	humanized neuroblastoma
Gorini et al. (24)	Nurse like cells	Selectively elimination of the CD1d-expressing M2-like macrophage population called nurse-like cells (NLCs) which sustain CLL cell survival	CD1d-iTCR	Chronic lymphocytic leukemia
Cortesi et al. (30)	Macrophages	Elimination of pro-tumor M2-like macrophages and support of anti-tumor M1-like ones	CD1d-iTCR CD40-CD40L Fas-FasL	Prostate cancer (TRAMP)
Janakiram et al. (88)	Macrophages	M2-like macrophages are increased in absence of iNKT cells	mPGES-1 5-LOX	LSL-KrasG12D/+ pancreatic cancer
Wang et al. (89)	Macrophages	In the absence of iNKT cells, shift from CD206^+^ M2 toward iNOS^+^ M1 macrophages, and a reduction of PMN-MDSC in polyps	–	ApcMin/+ model for colon cancer
Wang et al. (90)	Macrophages	M1-like switch of TAMs and activation of CD8 T cells	–	ApcMin/+ model for colon cancer

*Listed are the studies, the cells interacting with iNKT cells and the effect of these interactions, the molecules involved and the model analyzed*.

iNKT cells were first shown to inhibit MDSCs suppressive activity in a model of influenza A viral infection in a CD1d- and CD40- dependent manner (83). Several evidence support this ability also in cancer. iNKT cells promote the differentiation of MDSCs into functional DCs upon α-GalCer injection in a mouse model of colon carcinoma, leading to the activation of tumor-specific CD8^+^ and CD4^+^ T cells and the triggering of NK cell cytotoxicity (86). iNKT cells also reduce CD1d^+^ MDSCs numbers and activity (83, 87) enhancing the conversion of immature MDSCs to mature APCs (83, 86). In particular, in human melanoma patients, iNKT cells could also reverse the immune-suppressive activity of a population of neutrophils producing IL-10, by inducing their maturation toward IL-12-producing cells *via* CD1d- and CD40- interactions, ultimately reinstating the activation of tumor antigen-specific CD8^+^ T cells (84). These iNKT cell anti-tumor effector functions on immunosuppressive myelomonocytic cells in the TME are exerted by CD1d-cognate recognition. Observations in a humanized neuroblastoma mouse model highlight the elimination by the transferred human iNKT cells of CD1d-expressing TAMs, impairing their tumor-supporting activity (85). The importance of the iNKT cell-TAM crosstalk is further strengthened in the same humanized neuroblastoma model, by showing that iNKT cells are recruited in the TME tumor by CCL20-producing TAMs, but are progressively inhibited in their anti-tumor activity by macrophage-induced hypoxia (47). Notably, iNKT cells can discriminate between anti-tumor M1-like cells and pro-tumor M2-like ones, selectively eliminating pro-tumor M2-like macrophages while supporting anti-tumor M1-like populations (24, 30). For instance, in a model of oncogene-induced pancreatic cancer, M2-like macrophages are increased in absence of iNKT cells, suggesting the preferential targeting of this pro-tumor macrophage population by iNKT cells (88). By investigating at the same time a transgenic mouse model of CLL and patients with stable or progressive disease, we showed that iNKT cells delay the onset and progression of the leukemia by remodeling the supporting niche of the leukemia cells through the selective elimination of the CD1d-expressing M2-like macrophage population called nurse-like cells (NLCs) (24), which sustain CLL cell survival (24, 91). Furthermore, we have found that iNKT cells selectively restrict M2-like TAMs in the TME, delaying the progression of an autochthonous TRAMP mouse prostate adenocarcinoma model, while in human prostate cancer the disease aggressiveness correlates with reduced intra-tumor iNKT cells and increased M2 macrophages, underscoring the clinical significance of this crosstalk (30). Adoptively transferred iNKT cells were able to infiltrate the TME of TRAMP mice and selectively kill M2-like TAMs upon CD1d-stimulation and the combinatorial engagement of CD40 and Fas. Although both molecules are expressed to similar levels on either M1-like or M2-like TAM populations, *in vitro* co-culture experiments between mouse iNKT cells and M1- or M2- bone marrow-derived monocytes revealed that the CD40L-CD40 pathway support the selective survival of the M1 population, by antagonizing the apoptotic death driven by Fas signaling. By contrast, CD40 expression did not protect M2 cells from FAS-dependent killing, suggesting that CD40 engagement may transduce distinct intracellular signals between M1 and M2 macrophages. Interestingly, the iNKT ability to promote immunostimulatory Th1/M1-like conditions in the TME seems to be context-dependent. In fact, iNKT cells naturally promote the formation of polyps in the spontaneous murine adenomatous polyposis coli (Apc) ApcMin/+ model for colon cancer (89), associated with a shift from M1-like to M2-like TAMs and decreased pro-inflammatory/ T_H_1 associated gene expression, which were reverted in ApcMin/+ mice lacking iNKT cells (89). However, the treatment of ApcMin/+ mice with strong iNKT cell agonists (α-GalCer and C20:2) reduced the polyp size in the small intestine thanks to iNKT cell-dependent intra-tumor activation of CD8 T cells and M1-like switch of TAMs (90). Yet, iNKT cells showed reduced frequencies and PD-1 upregulation (90), suggesting an anergic state for iNKT cells in α-GalCer-treated mice (92, 93). Consistently, the addition of PD-1 blockade improved the treatment with the iNKT cell agonist α-GalCer and enhance anti-tumor activities, resulting in highly significant reduction of polyp numbers in the small and large intestine, maintenance of iNKT cells and a skew toward a T_H_1-like iNKT1 phenotype specifically in polyps (94). The dichotomous iNKT cell response in the different tumors may be related to changes undergoing in the TME: in healthy intestine NKT1 and NKT17 subsets are mostly represented, whereas as tumor progresses iNKT cells infiltrating intestinal polyps start to produce IL-10 (77).

Nevertheless, while on the one hand iNKT cells are emerging as potent mediators of cancer immune surveillance, on the other hand several mechanisms of tumor immune evasion from iNKT cell control are becoming increasingly clear. For instance, tumors can elicit the upregulating the inhibitory NK receptor Ly49C/F/H/I causing iNKT cell unresponsiveness, as reported in the TRAMP murine model (55). Of note, this dysfunctional iNKT cell phenotype could be rescued *in vitro* by simultaneous stimulation with α-GalCer and IL-12, which likely overrides the inhibitory signal. Furthermore, likewise T cells, also tumor-unresponsive iNKT cells were reported to express PD-1, and their responsiveness could be reverted by PD-1/PD-L1 blockade (93). Finally, additional mechanisms related to intra-tumor metabolic dysregulation, hypoxia or accumulation of toxic products could play a role in the induction of iNKT cell dysfunctions (95, 96). All these suppressive mechanisms impact the iNKT cell counts and T_H_1 cytokines production in patients with late-stage progressive disease and must be kept in mind for potential therapeutic applications.

Collectively, these data show that iNKT cells exert their main anti-tumor functions by primarily modulating different myelomonocytic cell populations in the TME.

## iNKT Cell Are Highly Suitable for Adoptive Immunotherapy of Cancer

Given that immunosuppressive cues derived from the TME represent the major hurdle that must be overcome by the current adoptive cell therapy strategies to become efficient, particularly in solid tumors (97, 98), the peculiar ability of iNKT cells to reprogram immunostimulatory conditions in the TME could be actively exploited to enhance the efficacy of the approach. Furthermore, iNKT cells possess other unique features that make them particularly suitable for adoptive immunotherapy of cancer. First, they are restricted for the monomorphic CD1d molecule, which is identical in all individuals permitting their functions across MHC barriers without risks of alloreactivity (99). Whereas not relevant in the autologous immunotherapy setting, GvHD is the major concern for adoptive cell therapy with allogeneic T cells, which are restricted for the polymorphic MHC molecules, and can give raise to alloreactive anti-host response by donor T cells. By contrast, iNKT cells have been shown in pre-clinical models to suppress GvHD and are associated with reduced GvHD in the clinic. Several studies have reported that GvHD is exacerbated in CD1d^−/−^ or Jα18^−/−^ mice and that stimulation of iNKT cells can increase anti-leukemia responses while simultaneously mitigating the severity of GvHD (100). Interestingly, in the context of hematopoietic stem cell (HSC) transplantation, preclinical and clinical studies demonstrate that iNKT cells significantly attenuate GvHD without abrogating the graft vs. leukemia (GvL) effect, exerted through direct and indirect mechanisms (99, 101–103). In pediatric acute leukemia patients, this process has been correlated with the engraftment of donor iNKT cells, as failure to reconstitute iNKT cells after transplantation strongly correlates with disease relapse (40). Studies into the mechanisms of GvHD suppression showed that iNKT cells modulate the overall grafted immune response through production of T_H_2 cytokines such as IL-4, which restrains inflammatory donor T cells, and promote T_reg_ proliferation against both acute and chronic GvHD (103). Adoptively transferred iNKT cells are at least 10 times more potent than T_regs_ in protecting mice from lethal GvHD without compromising the GvL effect (104). iNKT cells have the unique ability to secrete both T_H_1 and T_H_2 cytokines, in particular human CD4^+^ iNKT cells are able to secrete IL-4 and IL-13 whereas DN iNKT cells were able to secrete T_H_1 cytokines (15). Thus, T_H_2 iNKT cells facilitate the engraftment of allogenic donor cells against recurrence of leukemia, while the T_H_1 arm generate antitumor response (105).

Because of the lack of toxicity in the allogeneic setting, iNKT cells seem the ideal platform for the generation of “off-the-shelf” ready-to-use effector cells for adoptive immunotherapy of cancer. Nevertheless, allogeneic iNKT cells must be somewhat edited to downregulate their MHC expression to avoid a possible rejection mediated by the allogeneic host immune system. To this purpose, iNKT cells have been differentiated *in vitro* from human hematopoietic stem cells that express very low levels of HLA-I and almost undetectable HLA-II molecules, which then can be further engineered with CARs to generate stealth anti-tumor effectors for the host immune response (106). An alternative strategy to generate off-the-shelf allogeneic iNKT cells, currently being assessed in early phase clinical trials for patients with progressing B cell malignancies (ANCHOR NCT03774654), relies in the co-expression of CD19-CAR, IL-15, and shRNAs targeting beta-2 microglobulin and CD74 to downregulate surface HLA class I and class II molecules. Although the strategy, which is combined by the lymphodepletion of the recipients before iNKT cell transfer, shows promise, it should be kept in mind that the complete abrogation of MHC expression on the transferred allogeneic cells should trigger the “missing self” response by NK cells, ultimately eliminating the gene-edited iNKT cells. This may be mitigated by engineering the allogeneic tumor-redirected HLA-edited effectors to express ligands for the NK inhibitory receptors (107) or the “don't eat me signal” provided by CD47 to prevent phagocytosis by phagocytes (108), which can cooperate to promote long term effects of the adoptive cell therapy.

A possible pitfall for adoptive iNKT cell immunotherapy is that the median frequency of these cells in the human peripheral blood is 0.01% in most individuals and decreases further in advanced cancer patients. However, iNKT cells easily expand in culture and efficient protocols to activate and expand iNKT cells from the patients have been established (109). Considering the lack of histocompatibility barriers for iNKT cell functions, allogenic donor source could also be an intriguing experimental alternative. To overcome the issue, concerning the generation of a huge number of iNKT cells *in vitro* for clinical purpose, Taniguchi and colleagues developed an interesting strategy based on induced-pluripotent stem cells (iPSCs) technology. They were able to derive iPSCs from mouse splenic iNKT cells and to induce such high proliferative stem cells to differentiate into functional iNKT cells *in vitro*. The iPSC-derived iNKT cells recapitulated the adjuvant effects of physiologic iNKT cells and suppressed tumor growth *in vivo* (110). The same results were also obtained with human iPSC-derived iNKT cells, which could be activated by α-GalCer-pulsed DCs and produced as much IFNγ as natural parental cells but exhibited better cytotoxic activity against various tumor cell lines. iPSC-derived iNKT cells also possessed significant anti-tumor activity in tumor-bearing mice (111). In a recent work iNKT cells were also generated from human CD34^+^ HSC engineered to express the rearranged TCR genes from a iNKT cell clone (112). This study showed that HSC-iNKT cells have the expected properties of human iNKT cells in terms of their distribution, phenotype, and ability to secrete cytokines in a bone-marrow-liver-thymus (BLT) humanized mouse model. Moreover, *in vivo* HSC-iNKT cells could also protect against a multiple myeloma or a melanoma that expressed CD1d, without the requirement of α-GalCer, by the recognition of tumor-endogenous lipid antigens. iNKT cells have been actively exploited in several clinical studies, summarized in Table 2 (44, 106–113, 126).

**Table 2:** iNKT cells used in clinical studies on oncological patients.

**References**	**Trial ID**	**Tumor type**	***n***	**Treatment**
Giaccone et al. (113)	–	Solid tumors	24	αGalCer
Nieda et al. (114)	–	Metastatic solid tumors	12	αGalCer/immature MoDC
Nicol et al. (115)	–	Metastatic solid tumors	12	αGalCer/immature MoDC
Chang et al. (116)	–	Advanced cancer	5	αGalCer/mature MoDC
Ishikawa et al. (117)	UMIN000007321	NSCLC	11	αGalCer/immature DC enriched PBMC
Motohashi et al. (118)	–	NSCLC	6	αGalCer/IL-2-expanded PBMC
Uchida et al. (119)	–	Head and neck SCC	9	αGalCer /immature DC-enriched PBMC
Motohashi et al. (120)	–	NSCLC	23	αGalCer/immature DC-enriched PBMC
Kunii et al. (121)	–	Head and neck SCC	8	αGalCer/immature DC-enriched PBMC + αGalCer/IL-2-expanded PBMC
Yamasaki et al. (122)	UMIN000000852	Head and neck SCC	10	αGalCer-pulsed APCs + activated Vα24 NKT cell + surgery
Kurosaki et al. (123)	UMIN000001933	Head and neck SCC	17	a-GalCer/immature DC-enriched PBMC
Nagato et al. (124)		NSCLC	4	αGalCer /immature DC-enriched PBMC
Richter et al. (44)	NCT00698776	Multiple myeloma	6	αGalCer /mature MoDC + lenalidomide
Exley et al. (125)	NCT00631072	Advanced melanoma	*9*	*Ex-vivo* expanded iNKT cells
Gasser et al. (126)		Advanced melanoma	8	αGalCer + NY-ESO-1/mature MoDC
Heczey et al. (127)	NCT03294954	Neuroblastoma	24 (R)	Autologous GD2-CAR NKT cells expressing IL-15
–	NCT04754100	Multiple myeloma	30 (R)	agent-797 iNKT therapy
–	NCT03774654	B cell malignancies	48 (R)	Allogenic CD19-CAR NKT cells expressing IL-15
–	NCT04751786	Advanced Solid Tumor (NY-ESO-1^+^)	15 (R)	iNKT cell activator PRECIOUS-01 + NY-ESO-1 encapsulated in nanoparticles
–	NCT04814004	ALL, CLL, and B-cell lymphoma	20 (R)	Autologous CD19-CAR iNKT cells expressing IL-15

*Listed are the studies, the trial ID number when available, the tumor type, the number of patients involved and the type of treatment. NSCLC, non-small-cell lung cancer; SCC, squamous cell carcinoma; ALL, acute lymphoblastic leukemia; CLL, chronic lymphocytic leukemia; R, recruiting*.

In conclusion, iNKT cells are an attractive novel alternative to conventional T cells for cancer immunotherapy. Their CD1d restriction, tumor tissue tropism, ability to restrict the suppressive TME support their exploitation for advanced adoptive cell therapy to treat solid and hematological malignancies.

## iNKT Cells can be Engineered to Acquire a Second Antigen-Specificity

iNKT cells exert their anti-tumor effector functions by modulating in a CD1d-cognate recognition manner immunosuppressive myelomonocytic cells infiltrating the TME. Hence, considering the increased use of CAR- or TCR-engineered cells for adoptive cell therapy, it is likely that allogenic iNKT cells could be exploited as single effector for the dual targeting of cancer cells and suppressive tumor stroma, which is considered a critical factor for the efficacy of any adoptive cell therapy strategy. This approach implies that iNKT cells must utilize both their endogenous TCR and the exogenous tumor-specific CAR/TCR, unlike conventional T cells, in which the current tendency is to eliminate the expression of the endogenous TCRs to maximize the function of the transduced receptors. In fact, in an autologous setting, the endogenous TCRs expressed by a bulk polyclonal T cell preparations are unlikely to contribute to anti-tumor effects, while in an allogeneic setting, these TCRs would instead cause GvHD. Moreover, the expression of the endogenous TCR by CAR/TCR transduced iNKT cells provide another advantage over T cells, which is the ability to boosting their combinatorial anti-tumor functions with α-GalCer (or other agonists) *in vivo* and enhance the overall therapeutic effect of the engineered iNKT cells (128).

**Figure 1:**
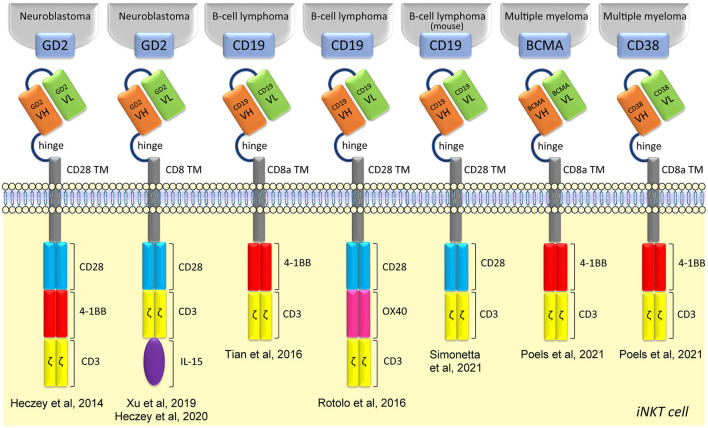
Structure of CAR iNKT-cells. CAR iNKT-cells are composed of several parts: an extracellular single-chain variable fragment (scFv), a hinge for flexibility and distance, a transmembrane spacer, different intracellular costimulatory molecules, and TCR CD3ζ subunit.

CAR therapies were first applied using conventional T cells, granting the approval by the Food and Drug Administration of two CAR-T cell therapies for acute lymphoblastic leukemia and for advanced lymphoma. Human iNKT cells can also be efficiently engineered to express GD2 CARs (against neuroblastomas), CD19 CARs (against B cell lymphomas), and CD38 or BCMA CARs (against multiple myeloma) (129–132), depicted in Figure 1. CAR-iNKT cells kill their relevant antigen-expressing tumor cell lines or patient-derived plasma cells *in vitro* (129–132) and tumor xenograft models *in vivo* (129–131), maintaining their CD1d-dependent functions. The lytic ability of CAR-iNKT cells appears independent of the costimulatory domain inserted in the CAR, whereas the CARs containing the 4-1BB domain seems to promote a better expansion capacity (132). GD2-CAR-iNKT cells persistence and anti-tumor activity can be further increased wit CAR constructs co-expressing human IL-15 (133), while increasing the expression of CD1d on B-lymphoma or leukemia cells with epigenetic drugs substantially enhanced their targeting by CD19-CAR-iNKT cells, resulting in markedly improved cancer control in mouse xenograft models (131). Notably, in this way, intravenously administered CAR19- iNKT but not CAR19-T-cells swiftly eradicated secondary brain lymphoma (131). Furthermore, in an immunocompetent mouse model of syngeneic B-cell lymphoma, CD19-CAR-iNKT cells exerted potent direct cancer cell killing and were also able to recruit host tumor-specific CD8 T-cell responses *via* facilitating tumor-antigen cross-priming, in turn enhancing long term cancer control (134). Phase I clinical trials with CAR-iNKT cells already showed promising results, in an interim analysis on children with relapsed or resistant neuroblastoma treated with autologous iNKT cells engineered to co-express a GD2-CAR with IL-15 (NCT03294954) (127). First, no dose-limiting toxicities were observed. Second, CAR-iNKT cells expanded *in vivo*, actively localized to tumor masses and, in one out of three patients, induced regression of bone metastatic lesions. Other two phase I clinical trials are exploring whether donor-derived or allogenic-(NCT03774654 and NCT04814004, respectively) iNKT cells transduced with CAR19 might help in patients with CD19^+^ lymphoma or leukemia.

iNKT cells can be also engineered to acquire a second antigen-specificity by expressing recombinant TCRs that recognize pathology relevant antigens and, in particular, tumor associated antigens. For instance, the transfer a human MHC-I restricted TCR specific for a peptide epitope derived from the Mycobacterium tuberculosis (Mtb) 38-kDa protein generated iNKT cells able to specifically kill Mo-DCs pre-loaded with the 38-kDa protein. The same TCR-engineered iNKT cells maintained the recognition of α-GalCer-pulsed Mo-DCs, suggesting that the endogenous iTCR was still fully functional (135). Human iNKT cells were also efficiently redirected against melanoma cell lines by engineering with a high affinity TCRs specific for an HLA-2-restricted peptide epitope derived from the tumor associated antigens (MART-1_26−35_, PRAME, Survin_96−104_). The TCR-iNKT cells efficiently killed antigen expressing melanoma cells *in vitro* and showed HLA-restricted antitumor activity in xenogeneic mouse models (136). However, in this study the iNKT cell endogenous TCR was completely displaced from the cell surface by the transferred TCRs, thus thwarting all the natural antitumor functions of iNKT cells. To overcome this limitation, there are several strategies that can be borrowed from TCR transfer in T cells to avoid the displacement of iNKT cell endogenous TCR, like modifications facilitating intramolecular bonding between transgenic α and β chains, fusing the chains to CD3ζ (137). Implementation of these strategies also for TCR transfer in iNKT cells will enable the generation of tumor retargeted cells which maintain, at the same time, the peculiar iNKT cells capacity of TME remodeling, having enhanced anti-tumor activities.

Notwithstanding all the above advantages of exploiting iNKT cell engineering for adoptive cell therapy of cancer, there also possible concerns connected with their use, for instance considering the prompt and abundant secretion of different cytokines by iNKT cells. The potent cytokine response produced by CAR-T cells upon target recognition *in vivo* is in fact responsible for the serious acute systemic toxicity (particularly neurological) often observed in treated patients. We currently do not know whether this side effect may reduce, or increase, when using CAR-iNKT cells. A recent study reported that iNKT cells transiently expressing a RNA-based anti-CSPG4 CAR produced much lower quantities of IL-6 and other cytokines involved in cytokine release syndrome (i.e., TNF and IFNγ) than the CAR-transfected CD8^+^ T cells, even if they have equal specific cytotoxicity (138). Nevertheless, the results of the ongoing CAR-iNKT cell clinical trials will clarify the actual safety and toxicity profiles of this approach, compared to CAR-T cells.

CAR- and TCR-iNKT cells therefore represent a potential new generation of dual-specific effector cells that warrant additional investigation to assess their anti-tumor efficacy in adoptive cell therapy, compared to T cells. This paves the way for advanced cell therapies in cancer patients, free from the concerns for HLA matching and possibly exploiting a redirected specificity toward tumor associated antigens combined with the TME remodeling exerted by the unique iNKT cell population.

## Author Contributions

GD, PD, GC, and MF selected the review topic, collected relevant publications, and wrote the review. All authors contributed to the article and approved the submitted version.

## Funding

The study was funded by a FIRC-AIRC Fellowship Number 2019-22604 to GD, Grants from Fondazione Cariplo 2018-0366 to MF, Worldwide Cancer Research 19-0133 to GD, Associazione Italiana Ricerca sul Cancro (AIRC) IG2017-ID.20081 to GC, Italian Healthy Ministry project on CAR T RCR-2019-23669115 to GC and PD, and Fondazione AIRC under-5-per-Mille 2019-ID.22737 to PD.

## Conflict of Interest

The authors declare that the research was conducted in the absence of any commercial or financial relationships that could be construed as a potential conflict of interest.

## Publisher's Note

All claims expressed in this article are solely those of the authors and do not necessarily represent those of their affiliated organizations, or those of the publisher, the editors and the reviewers. Any product that may be evaluated in this article, or claim that may be made by its manufacturer, is not guaranteed or endorsed by the publisher.
